# The natural history of varicella zoster virus infection in Norway: Further insights on exogenous boosting and progressive immunity to herpes zoster

**DOI:** 10.1371/journal.pone.0176845

**Published:** 2017-05-18

**Authors:** Luigi Marangi, Grazina Mirinaviciute, Elmira Flem, Gianpaolo Scalia Tomba, Giorgio Guzzetta, Birgitte Freiesleben de Blasio, Piero Manfredi

**Affiliations:** 1 Department of Economics and Management, University of Pisa, Pisa, Italy; 2 Department of Infectious Diseases Epidemiology and Modeling, Infection Control and Environmental Health, Norwegian Institute of Public Health, Oslo, Norway; 3 Mathematics Department, University of Rome Tor Vergata, Rome, Italy; 4 Kessler Foundation, Trento, Italy; 5 Oslo Centre for Biostatistics and Epidemiology, Department of Biostatistics, Institute of Basic Medical Sciences, University of Oslo, Oslo, Norway; Katholieke Universiteit Leuven Rega Institute for Medical Research, BELGIUM

## Abstract

We use age-structured models for VZV transmission and reactivation to reconstruct the natural history of VZV in Norway based on available pre-vaccination serological data, contact matrices, and herpes zoster incidence data. Depending on the hypotheses on contact and transmission patterns, the basic reproduction number of varicella in Norway ranges between 3.7 and 5.0, implying a vaccine coverage between 73 and 80% to effectively interrupt transmission with a 100% vaccine efficacy against infection. The varicella force of infection peaks during early childhood (3–5 yrs) and shows a prolonged phase of higher risk during the childbearing period, though quantitative variations can occur depending on contact patterns. By expressing the magnitude of exogenous boosting as a proportion of the force of infection, it is shown that reactivation is well described by a progressive immunity mechanism sustained by a large, though possibly below 100%, degree of exogenous boosting, in agreement with findings from other Nordic countries, implying large reproduction numbers of boosting. Moreover, magnitudes of exogenous boosting below 40% are robustly disconfirmed by data. These results bring further insight on the magnitude of immunity boosting and its relationship with reactivation.

## Introduction

The varicella zoster virus (VZV) is transmitted by droplets, aerosols, and direct contact, and is responsible for both varicella (chickenpox) and herpes zoster (HZ). The former is a highly transmissible, usually mild, infection acquired early in life [1], with 75–95% of European children being immune by age 12 in the absence of vaccination [2]. Complications can occur in 2%-6% of cases [3]. After recovery from varicella, VZV remains latent in nerve ganglia and can reactivate at later ages causing HZ, a painful skin disease responsible for serious morbidity, as the most often reported post-herpetic neuralgia [4]. Although HZ immunology and pathogenesis are complex and still poorly understood, after Hope-Simpson seminal study attributing reactivation to a decline in antibodies [5], consensus has emerged that HZ arises when cell-mediated immunity (CMI) declines, for example as a consequence of senescence [4]. Hope-Simpson [5] also hypothesized that immunity against HZ might be boosted *exogenously* by further exposures to VZV. The exogenous boosting hypothesis (EBH) was investigated in later studies and some supporting evidence gradually accumulated [6–10], though the actual magnitude of boosting is still poorly assessed [10]. Nonetheless, the largest available European serological study on VZV has indicated a lower age-specific HZ incidence in countries where the force of infection of varicella is higher [2].

At present, despite the availability of a safe and effective vaccine since 1970s [11], the introduction of varicella immunization in Europe is halted, with only a few European countries having introduced the vaccine into their national programs [12], due to feared negative effects of varicella vaccination on the HZ incidence.

Indeed, after the seminal work in [13], essentially all mathematical models for VZV transmission and reactivation relying on the EBH have predicted an increase in the natural incidence of HZ for several decades following the introduction of a mass varicella vaccination [14–21,9]. This is due to the fact that, under the EBH, effective varicella immunization programs would also reduce the incidence of re-exposure to a wild VZV, thereby removing this natural protection against HZ. Summing up, the presence and magnitude, of exogenous boosting currently represent the critical factors which might make varicella vaccination not being cost-effective [19]. In relation to this, a multi-country study [18] has showed that milder effects of varicella vaccination on HZ arise when the magnitude of exogenous boosting intensity is estimated to be low.

In Norway, where the varicella vaccine is currently not offered through the national immunization program, and the number of those immunized annually is negligible, the first large scale serological study on VZV has recently been conducted, providing information about acquisition of varicella infection [22]. Moreover, detailed age-stratified HZ incidence data are available [22].

Mathematical models of infection transmission (and sequelae), and their parameters, such as the force of infection and the basic reproduction number (R_0_), represent efficient tools to combine available data in order to reconstruct the underlying natural history of infection [23,24,6,2].

This paper uses age-structured models for VZV based on available pre-vaccination serological data [22], contact matrices [25], and herpes zoster incidence data [22], with a three-fold aim i.e., (i) to provide the first model-based reconstruction of the natural history of VZV in Norway, (ii) to estimate critical epidemiological parameters that will be taken as primary inputs for future mathematical models assessing immunization strategies against varicella and HZ, and (iii) to bring fresh evidence about the role of exogenous CMI boosting in shaping HZ incidence profiles. To address the latter we devote special attention to characterising the magnitude of exogenous boosting, and propose the use of a specific reproduction number to summarise the intensity of the boosting phenomenon.

## Methods

### Data

Norwegian VZV sero-prevalence data included 2103 anonymised residual sera from patients of all ages seeking both primary and hospital care during 2006, 2007, 2008, 2011 and 2014. Samples collected during the H1N1 2009–2010 influenza pandemic were deliberately excluded, as well as the very few samples available from 2012 to 2013 [22].

As for age-specific HZ incidence, we used estimates from HZ-associated consultation rates in patients attending primary healthcare in Norway.

As regards social contact patterns, we used both the synthetic contact matrix for Norway computed elsewhere [25], where age-specific contact matrices were reconstructed for all European countries by combining official socio-demographic data and individual-based simulation, as well as some questionnaire-based contact matrices from the Polymod study [26] for Finland, another Nordic country sharing a similar population and socio-economic profile. For the synthetic contact matrix we used the overall matrix, including contacts from all different settings (households, school and workplaces, and the general community), while for the “Polymod” matrices we considered both the overall matrix for all reported contacts, as well as the matrix for “close” (i.e., physical contacts of long duration) contacts, used in a number of studies because of its high quality fit to varicella serological data [27–28]. We denote the alternative types of matrices considered as “synthetic”, “Polymod-all”, and “Polymod-close”, respectively.

Demographic data, namely age-specific fertility and mortality rates, were provided by Eurostat [29]. Information on the Norwegian school system and school enrolment rates were obtained from Norwegian national sources [30–31].

### Mathematical model

**Fig 1:**
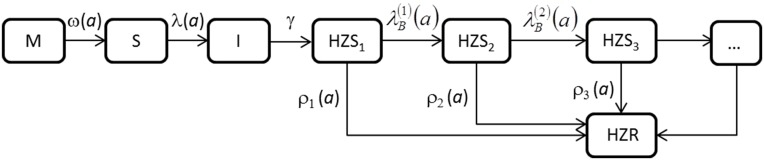
Flow diagram of the compartmental model for VZV transmission and reactivation. Boxes denotes epidemiological compartments (M = maternal antibody, S = susceptible to varicella infection, I = varicella infective, HZS_1_ = first time zoster susceptible, etc.), while arrows denote transfers between compartments; ω(a) denotes the rate of loss of maternal antibodies, λ(a) the varicella force of infection (FOI), γ the varicella recovery rate, $\lambda_{B}^{\left(h\right)}\left(a\right)$ (h = 1,2,3…) the force of exogenous boosting (FOB), and ρ_i_(*a*) (i = 1,2,3…) the rates of HZ development in each HZ susceptibility stage.

The age-structured model for the natural history of VZV transmission and reactivation in Norway (Fig 1, details in S1 File), comprises an MSIR sub-model for varicella transmission, plus a cascade of further compartments to account for the different episodes of exogenous boosting. The model rests on the following hypotheses [32]: (a) the population is at demographic equilibrium with a constant number of births per year and a time-invariant age-specific mortality rate μ(*a*), where *a* denotes chronological age; (b) VZV is at its endemic pre-vaccination equilibrium, as motivated by the low number of vaccines administered in Norway per year [22], implying that the age-specific force of infection (FOI) with varicella and the force of boosting (FOB) are time-independent; (c) the contribution of active zoster cases to FOI and FOB is assumed to be negligible, as supported by the literature [33,15]. This allows the varicella transmission part of the model to decouple from the zoster part.

In particular, newborn individuals [34] are either protected by maternally acquired antibodies (compartment M) or susceptible to varicella infection (S), depending on whether they were born to an immune or a susceptible mother; M-type individuals are protected by maternal antibodies for a fixed duration D_M_ after which they become susceptible to varicella; susceptible individuals acquire varicella infection from contacts with infective individuals at the age dependent force of infection λ(*a*), becoming infective (I); infective individuals recover at constant rateγ; varicella recovered individuals (traditionally denoted by R) are permanently immune to varicella but at the same time become susceptible to HZ (stage HZS_1_); individuals in stage HZS_1_ can either be re-exposed to VZV, through infectious contacts, thereby receiving an exogenous CMI boosting at a force of boosting *$\lambda_{B}^{\left(1\right)}\left(a\right)$*, or develop HZ with stage-specific reactivation rate ρ_1_(*a*, τ), where τ (τ≤*a*) represents the duration spent in the stage [16,32]. Re-exposed individuals enter HZ susceptibility stage HZS_2_ where again they can either be exogenously boosted at rate $\lambda_{B}^{\left(2\right)}\left(a\right)$ (moving to HZS_3_) or develop HZ at rate ρ_2_(*a*, τ) and so on. Individuals who have experienced HZ are lifelong immune to HZ (compartment HZR) owing to the evidence that the lifetime risk of a second HZ episode is only between 1% and 5% among immuno-competent individuals [4]. The MSIR structure was adopted to account for the susceptible proportion among newborns, given the quite large proportion of susceptible people during the childbearing period documented by serological data [22].

### Varicella force of infection, contact patterns and varicella transmission patterns

By the hypothesis that the contribution of active HZ cases to the varicella FOI is negligible, the age-specific FOI of varicella in age group i at endemic equilibrium is defined as $\lambda_{i}=\Sigma_{j\in G}q_{ij}C_{ij}\;\frac{I_{J}}{N_{J}}$, where G represents the adopted age grouping, C_ij_ is the average number of contacts an individual aged i has with individuals aged j per unit of time (e.g. per day), q_ij_ is the varicella age-specific transmission rate per single social contact between a susceptible aged i and an infective aged j, and *I*_*j*_*/N*_*j*_ is the endemic infective prevalence at age j, given by the ratio between the number of infective individuals and the population in that age group. We considered the following age-grouping (in completed years): 0, 1–2, 3–5 (preschool), 6–12 (primary school), 13–15,16–18, 19–25, and 5-years age bands thereafter, plus an 80+ class. This age grouping reflects the structure of the Norwegian school system and the observed differences in age-specific enrolment rates in organized childcare (3,6% at age 0, 80.1% at age 1–2 y, 96.7% at age 3–5 y) [30].

As for the age-specific transmission rates q_ij_ we considered both constant (i.e., age-independent) transmission: q_ij_ = q, known as the social contact hypothesis [35], and a number of parsimonious age-dependent formulations. The latter are described by the following discrete 2-parameters transmission matrices Q_1_,…, Q_5_ [27], amounting to partition transmission into large age-blocks based on some cut-off age *a*_c_:

$$Q_{1}=\left(\begin{array}{cc} q_{1} & q_{2}\\ q_{2} & q_{2} \end{array}\right);\;Q_{2}=\left(\begin{array}{cc} q_{1} & q_{1}\\ q_{2} & q_{2} \end{array}\right);\;Q_{3}=\left(\begin{array}{cc} q_{1} & q_{2}\\ q_{2} & q_{1} \end{array}\right);\;Q_{4}=\left(\begin{array}{cc} q_{1} & 0\\ 0 & q_{2} \end{array}\right);\;Q_{5}=\left(\begin{array}{cc} q_{1} & q_{2}\\ q_{1} & q_{2} \end{array}\right);\;Q_{0}=\left(\begin{array}{cc} q & q\\ q & q \end{array}\right);$$

where we have denoted by Q_0_ the case of constant transmission (q_1_ = q_2_ = q). In the literature the cut-off age *a*_c_ has been typically set *a priori* (e.g., [27]). In this work we instead considered it as a further parameter.

### Force of (exogenous) immunity boosting

As for the FOB, we investigated both the traditional *full-boosting* case $\lambda_{B}^{\left(h\right)}\left(a\right)=\lambda \left(a\right)$ (h = 1,2,…), where each re-exposure boosts CMI at a rate identical to the force of first infection, and the *incomplete boosting* case: $\lambda_{B}^{\left(h\right)}\left(a\right)=z\lambda \left(a\right)$, where the *boosting constant z* (0<z<1) is a further parameter to be estimated from data.

### Reproduction numbers

The basic reproduction number of an infection, denoted by R_0_, is defined as the number of secondary infections caused by a single "typical" infective case (during his entire infective period) in a wholly susceptible population [36], and is computed as the dominant eigenvalue of the ‘‘next generation” matrix NG having elements NG_ij_ = Dq_ij_C_ij_, where D is the average duration of the infective phase. The effective reproduction number R_E_ extends this concept to an arbitrary distribution of susceptibility by age. In particular, at endemic equilibrium R_E_ is computed as the dominant eigenvalue of the matrix NG_ij_ (S_i_/N_i_) where S_i_/N_i_ is the endemic susceptible fraction in age group i, and we expect the equality R_E_ = 1 to hold, corresponding to the intuition that the typical infective case produces on average one new infection case. The value of R_E_ produced by the fitted model therefore represents a check of the validity of the adopted model and of the underlying assumptions [28]. To assess the importance of exogenous boosting we used episode-specific and overall reproduction numbers of boosting, quantifying the number of boosts produced by a typical varicella infective individuals during his infective period at endemic equilibrium. Episode-specific reproduction numbers of boosting are computed as the dominant eigenvalues of the matrices of elements $z\cdot NG_{ij}\left(ZS_{i,h}^{e}/N_{i}\right)$ where $ZS_{i,h}^{e}/N_{i}$ represents the endemic proportions of HZ susceptible aged i in HZ susceptibility stage h (h = 1,2, etc).

### Reactivation

As for the stage-specific risk of HZ development ρ_i_(a, τ) (that we also term reactivation rate, RR), we considered a number of alternative biological hypotheses (see Table 1). These hypotheses can be concisely described as sub-cases of Hope-Simpson’s progressive immunity hypothesis [32], according to which the RR is exponentially increasing in both (i) chronological age (beyond some threshold age *a*_0_), mimicking a senescence effect, and in (ii) the time τ elapsed since last exposure to VZV, mimicking a direct boosting effect, while it is exponentially declining in the cumulative number of boosting events the individual has experienced. The corresponding expression of the RR is as follows: *ρ*_*i*_(*a*, *τ*) = *ρ*_0_ ⋅ *R*_*a*_ ⋅*R*_*τ*_ ⋅ *R*_*i*_, where (i) *ρ*_0_ is a scale factor, (ii) $R_{a}=e^{\vartheta_{a}{\left(a-a_{0}\right)}_{+}}$ is the exponential component along chronological age, where (*a* − *a*_0_)_+_ = max(*a* − *a*_0,_0) and *ϑ*_*a*_ is the corresponding growth rate, (iii) $R_{\tau }=e^{\vartheta_{\tau }\tau }$ is the exponential component (at rate *ϑ*_*τ*_) along time elapsed since the last boosting episode, (iv) $R_{i}=g^{{\left(i-1\right)}^{2}}$ is the progressive immunity component where g (0<g<1, i = 1,2,…) is the progressive immunity factor. For g = 1 the RR becomes independent of the number of exposure episodes i (i.e., ρ_1_ = ρ_2_ = … ρ), obtaining the model used in [16], termed “baseline” in the sequel. By further setting *ϑ*_*τ*_ = 0, one gets a Gompertz-like model, where the RR is only senescence related, while for *ϑ*_*a*_ = 0 the RR depends only on the time elapsed since the last exposure to VZV (“duration since exposure”). The threshold age *a*_0_ aims to capture the rapid increase in HZ incidence beyond a certain age observed in available datasets [32], including Norway [22]. Here, instead of setting *a*_0_ a priori as in [19,32], we run a preliminary model selection analysis (details in ESM), which showed that only values in a narrow window around *a*_0_ = 40 yrs were compliant with Norway HZ data. We therefore set *a*_0_ = 40 as the reference value.

**Table 1:** The different hypotheses about the VZV reactivation rate.

Components of the reactivation rate →	Chronological age	Duration since exposure	Progressive immunity	Model parameters
	$R_{a}=e^{\vartheta_{a}a}$	$R_{\tau }=e^{\vartheta_{\tau }\tau }$	$R_{i}=g^{{\left(i-1\right)}^{2}}$	
Progressive immunity model [32]	Y	Y	Y	(*ρ*_0_, *ϑ*_*a*_, *ϑ*_*τ*_, *g*)
Gompertz (Age-senescence only)	Y	N	N	(*ρ*_0_, *ϑ*_*a*_)
Duration since (last) exposure	N	Y	N	(*ρ*_0_, *ϑ*_*τ*_)
Baseline model [16]	Y	Y	N	(*ρ*_0_, *ϑ*_*a*_, *ϑ*_*τ*_)
Gompertz and progressive immunity	Y	N	Y	(*ρ*_0_, *ϑ*_*a*_, *g*)
Duration and progressive immunity	N	Y	Y	(*ρ*_0_, *ϑ*_*τ*_, *g)*

The different hypotheses considered about the functional form of the RR as expressed by the presence (Y) or absence (N) of the exponential terms *R*_*a*_, *R*_*τ*_, *R*_*i*_, respectively tuning the components of risks along (a) chronological age, (b) duration elapsed since the last re-exposure event, and (c) number or re-exposure episodes.

### Models fit to serological and zoster incidence data

The different models considered were fitted to data in two sequential stages [32]. In the first stage varicella transmission parameters q’s were estimated by maximisation of the Bernoulli log-likelihood of serological data [35] based on an MSIR sub-model for varicella transmission at endemic equilibrium. Thanks to the hypothesis that the contribution of HZ to the varicella FOI is negligible, the transmission sub-system decouples, allowing to estimate transmission parameters by a simpler model not including HZ compartments. A large number of transmission models was then fitted to data, in order to consider different types of contact matrices (the synthetic one vs the two types of Polymod matrices), different hypotheses on transmission (age-dependent or not), and different values of the cut-off age *a*_c_ in the age-grid defined by the boundaries of the school age groups, namely exact ages 1,3, 6, 13, etc.

In the second stage, reactivation parameters (*ρ*_0_, *ϑ*_*a*_, *ϑ*_*τ*_, *g*) were estimated by maximising the Poisson log-likelihood of counts of HZ cases (per 5-years age groups), based on the best estimates of transmission parameters (and related uncertainty) obtained during the first stage.

As for exogenous boosting, in order to avoid overfitting problems and correlations between reactivation parameters and the boosting constant z, we investigated the behavior of the estimates of reactivation parameter over a thin grid of values of the boosting constant *z* on its admissible interval [0,1].

Whenever relevant, e.g., when comparing distinct age-dependent transmission models based on the same contact matrix, model selection was based on the Akaike information criterion [37] corrected for small sample size (AIC_*C*_). Nonetheless, we deliberately not use model selection measures when comparing models based on different contact matrices, in order to avoid to miss the underlying structural differences in contact patterns.

Uncertainty about unknown parameters was estimated by parametric bootstrap percentile confidence intervals (PCI).

## Results

### Transmission, force of infection and basic reproduction number of varicella

For each of the three contact matrices considered (synthetic, Polymod-all, Polymod-close) we report the estimates for the case of age-independent transmission (Q_0_), as well as the estimates for the age-dependent formulations (Q_1_,…, Q_5_) over the possible choices of the cut-off age *a*_c_. In particular, for each model included, we report (Table 2) the estimates of transmission parameters q_ij_, the AIC score, and the related estimates of the basic and effective reproduction numbers (R_0_ and R_E_), and of the average age at infection (A_I_). Note preliminary that the effective reproduction number (R_E_) at endemic equilibrium equals one up to the fifth digit for all models, an indirect confirmation of the goodness of the hypotheses made. The main substantive facts are as follows. First, among models with age-independent transmission (model Q_0_), only the one based on the Polymod matrix of close contact reproduces the data satisfactorily in statistical terms. Inclusion of age-dependent transmission always improves the fit, with Polymod-type matrices of close contacts tending—as a rule—to perform better than other matrices. The basic reproduction number (R_0_) of varicella ranged between 3.7 and 5.0 among the different models considered, implying the critical coverage by a 100% efficacious and lifelong vaccine, given by p_c_ = 1-(1/R_0_) [23], to range between 73 and 80.0%. Last, the average age at varicella infection ranged between 6.6 and 7.7 yr.

**Table 2:** Summary results for ML estimation of MSIR transmission models.

Contact matrix	Transmission model (Q) and cutoff age *a*_c_	q_1_	q_2_	AIC_C_	R_0_	R_E_	A_I_
Synthetic	Q_0_	0.08942	--	888.8	5.01	1.00002	7.73
Synthetic	Q_1_, a_*c*_ = 16	0.10098	6.05E-02	881.1	4.05	1.00003	6.92
Synthetic	Q_3_, *a*_c_ = 16	0.10097	5.52E-02	881.2	5.01	1.00003	6.93
PF-all	Q_0_	0.05469	--	892.4	4.79	1.0001	7.67
PF-all	Q_1_, *a*_c_ = 6	0.08920	5.16E-02	878.6	4.56	1.0002	7.034
PF-all	Q_2_, *a*_c_ = 6	0.08226	5.14E-02	878.4	4.68	1.0002	7.030
PF-close	Q_0_	0.11026	--	880.8	3.79	1.0001	7.46
PF-close	Q_3_, *a*_c_ = 16	0.12371	6.96E-02	876.1	3.75	1.0002	6.62

The natural history of VZV in Norway. Summary results of ML estimation for MSIR transmission models for varicella. Column 1: the adopted contact matrix i.e., synthetic vs the two types of Polymod matrices (from Finland), denoted as PF-all, and PF-close, respectively. Column 2: the adopted transmission model (among hypotheses Q_0_,…, Q_5_) and related cut-off age *a*_c_. Column 3,4: ML estimates of related transmission parameters q_ij_. Column 5: values of the corrected Akaike information criterion (AIC_C_). Column 6,7: related ML estimates of varicella basic (R_0_) and effective (R_E_) reproduction numbers. Column 8: best estimate of the average age at infection (A_I_).

**Fig 2:**
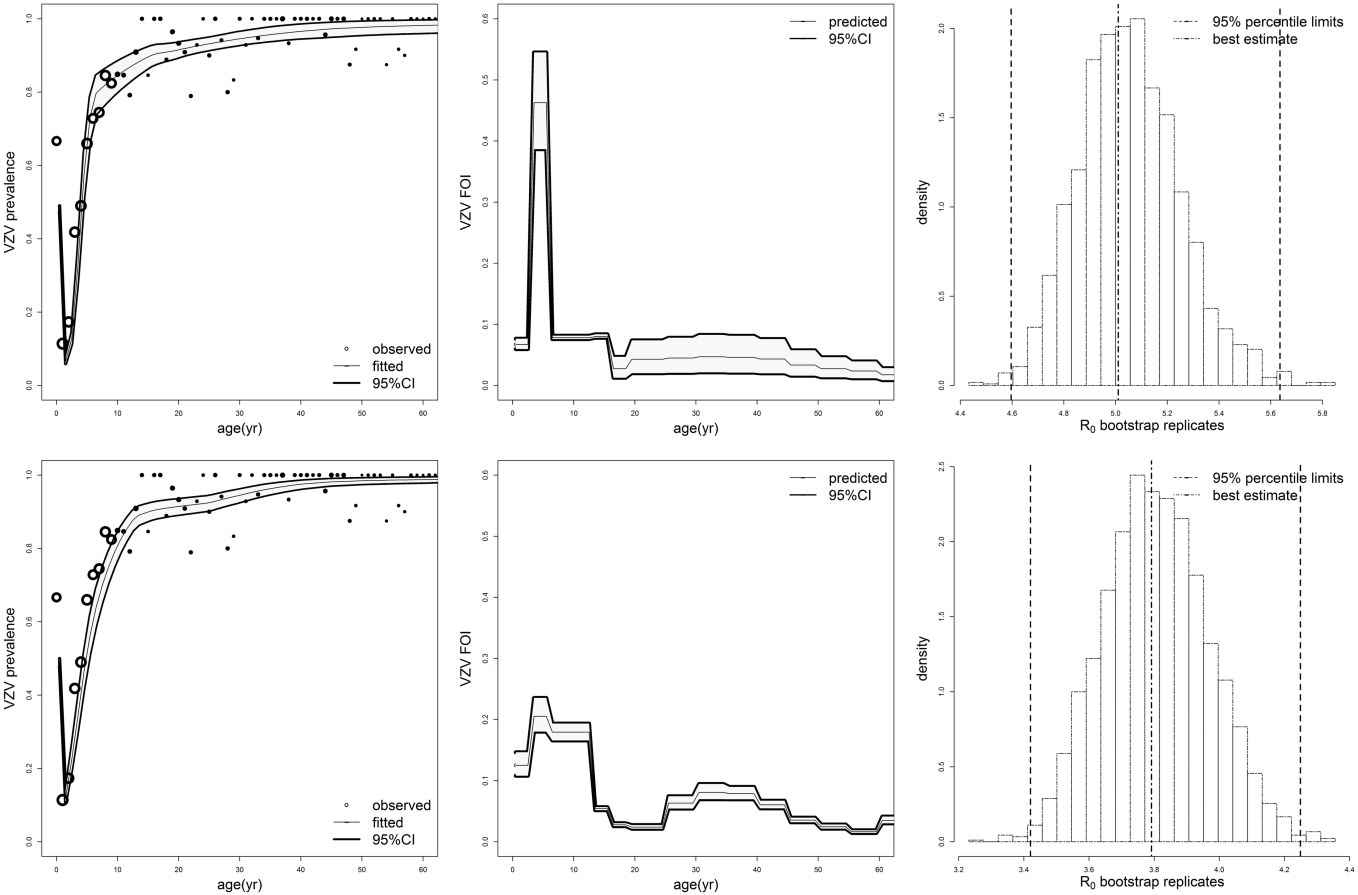
The natural history of VZV in Norway: Fit to seroprevalence data and force of infection. Top row: results from model Q_3_,16 based on the synthetic matrix; bottom row: results from model Q_0_ based on the Polymod matrix of close contacts. Left: predicted vs observed varicella age-specific sero-prevalence, with 95% confidence bands. Center: predicted age-specific varicella FOI with 95% confidence bands. Right: best R_0_ estimate, related distribution of bootstrap estimates and 95% percentile CI. For sake of comparison between models based on synthetic matrices (based on census data, and therefore lacking an uncertainty evaluation) vs models based on Polymod matrices (based on survey data), the bootstrap was done by re-sampling sero-prevalence data only.

Implications of previous results are displayed (Fig 2) for two of the models reported in Table 2, namely model Q_3_,_16_ based on the synthetic matrix (top row), and the age-independent transmission model Q_0_ based on the Polymod matrix for close contacts (bottom row). Though the reproduction of VZV sero-prevalence data appears satisfactory (Fig 2, left panels), there are remarkable quantitative differences in the age-profile of the FOI (Fig 2, center), clearly due to the differences in the underlying contact matrices. The synthetic matrix yields a marked peak during the preschool period (ages 3–5 years), it suddenly declines thereafter, and shows a new increase with a prolonged phase of relatively higher FOI lasting the entire childbearing period. For the Polymod matrix the peak is still located during pre-school ages but it is much lower and the FOI persists at a high level during the entire primary school (age 6–12 years); moreover the childbearing peak is more concentrated and more well-defined. These differences are the consequence of the known structural differences between the two types of matrices [25], which will be worth considering in future mathematical modelling of impact of vaccination. Overall, Table 2 and Fig 2 suggest that a there is range of models capable to fit varicella transmission data similarly well, yet these model can yield a substantial variation in fundamental VZV parameters (such as R_0_ and the FOI), therefore suggesting the existence of model-based uncertainty.

The varicella incidence predicted by the models in Fig 2 is reported in Table 3 for broad age classes.

**Table 3:** Predicted age-specific varicella incidence.

Age	0	1–2	3–5	6–15	16–24	25–44	45+
Synthetic, Q3, *a*_c_ = 16	4286	6867	20658	921	409	234	42
PF-close, Q_0_	6661	10694	12745	4197	297	268	33

The natural history of VZV in Norway. Age-specific incidence of varicella (per 100.000, per year) as predicted from the two transmission models reported in Fig 2.

### VZV reactivation and the role of exogenous boosting

**Fig 3:**
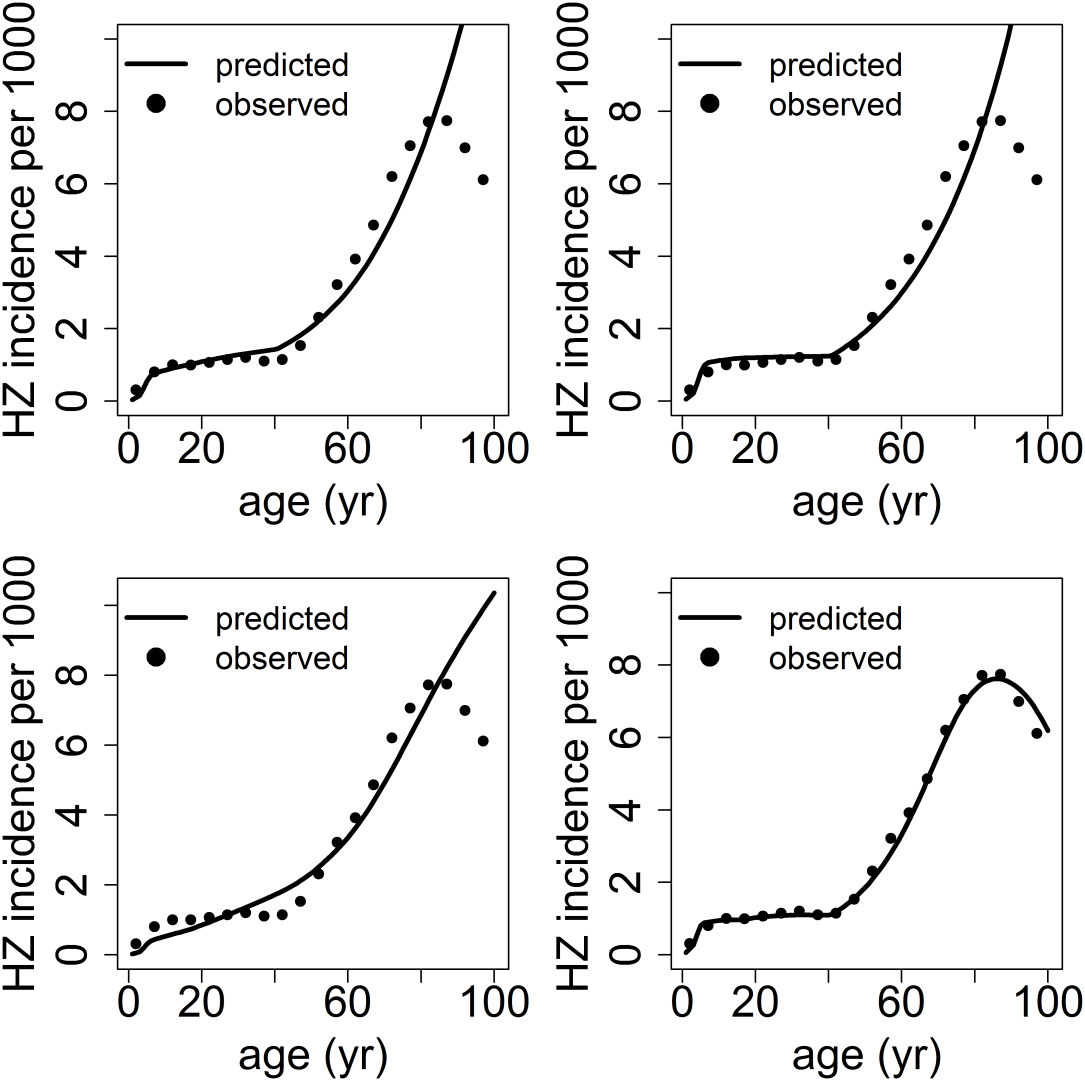
The natural history of VZV in Norway: Fit to age-specific HZ incidence by some models for VZV reactivation (Table 1) under the full boosting hypothesis (z = 1). Top-left: the “duration since exposure” model; top-right: the Gompertz age-dependent model; bottom-left: the baseline model; bottom-right: the progressive immunity model. The underlying transmission model is based on the synthetic matrix, hypothesis Q_3_, 16. HZ incidence is represented per 1000 population, per year.

Following the adopted two stage procedure, the alternative models for the RR (Table 1) were fitted to HZ incidence data based on each of the transmission models reported in Table 2, by retaining only the models which additionally showed a good fit to HZ. The results indicate that the progressive immunity model systematically out-performs the other models considered for both the traditional full-boosting case (z = 1, Table 4), as well as the imperfect boosting case (ESM), regardless of the hypotheses about transmission. Considering, for the sake of illustration, the progressive immunity model based on the synthetic matrix Q_3,16_ (Table 4), we see that the estimate of the rate (*ϑ*_τ_) of the duration-since-last-boost component is about 3,7%/year, while the corresponding rate of the senescence-related component (*ϑ*_*a*_) is about 5,7%/year. This means that, other things kept constant (i.e. in absence of further boosts), the RR faced by a person aged less than 40 years of age will increase at a rate of 3.7% per year, while it will increase at a yearly rate of about (3.7+5.7) = 9.4% for a person above 40 years. On the other hand, the estimated progressive immunity factor of about 0.59 means that, other things kept constant, the relative risk of reactivation for a person boosted once is about 59% compared to a never-boosted person, while it declines to (0.59)^2^, that is less than 35%, for a person boosted twice. Note in particular that (Fig 3) the progressive immunity model is the only one capable of capturing the overall shape of the HZ incidence profile, i.e. it does not simply capture, unlike other models, the declining incidence above age 80, but it is also more effective in modulating both the rapid growth at ages 40–60 and the subsequent slowing-down and plateauing. Indeed the progressive immunity model performs better than alternative models even when the fit is run excluding the very old, i.e. considering ages 0–84 only, as also shown in [32] (further details in ESM).

**Table 4:** ML estimation of the reactivation risk: The full-boosting case (z = 1).

Reactivation Model & fitted parameters	Underlying transmission model	ML estimates	AIC
Progressive Immunity (*ρ*_0_, *ϑ*_*a*_, *ϑ*_*τ*_, *g*)	Synthetic, Q_0_	(0.00129; 0.06339; 0.04966; 0.71241)	137.9
	PF-close,Q_0_	(0.00153; 0.06288; 0.04726; 0.61808)	139.4
	Synthetic, Q_1_, 16	(0.00161; 0.05535; 0.03883; 0.59800)	139.0
	Synthetic, Q_3_, 16	(0.00166; 0.05733; 0.03723; 0.59245)	139.3
Baseline (*ρ*_0_, *ϑ*_*a*_, *ϑ*_*τ*_)	Synthetic, Q_0_	(0.00110; 0.03441; 0.02184)	214.8
	PF-close,Q_0_	(0.001066; 0.03146; 0.02328)	235.3
	Synthetic, Q_1_, 16	(0.00089; 0.02602; 0.03049)	208.0
	Synthetic, Q_3_, 16	(0.00091; 0.02781; 0.02882)	209.0
Gompertz (*ρ*_0_, *ϑ*_*a*_)	Synthetic, Q_0_	(0.00132; 0.04716)	212.4
	PF-close,Q_0_	(0.001339; 0.04730)	213.6
	Synthetic, Q_1_, 16	(0.001332; 0.04780)	217.2
	Synthetic, Q_3_, 16	(0.00134; 0.04764)	216.8
Duration since exposure (*ρ*_0_, *ϑ*_*τ*_)	Synthetic, Q_0_	(0.00047;0.08230)	236.7
	PF-close,Q_0_	(0.00058;0.06927)	235.2
	Synthetic, Q_1_, 16	(0.00050; 0.06504)	223.6
	Synthetic, Q_3_, 16	(0.00048;0.06668)	226.0

The natural history of VZV in Norway under the full-boosting hypothesis (z = 1): parameter estimates and model selection measures for some of the reactivation models in Table 1

**Fig 4:**
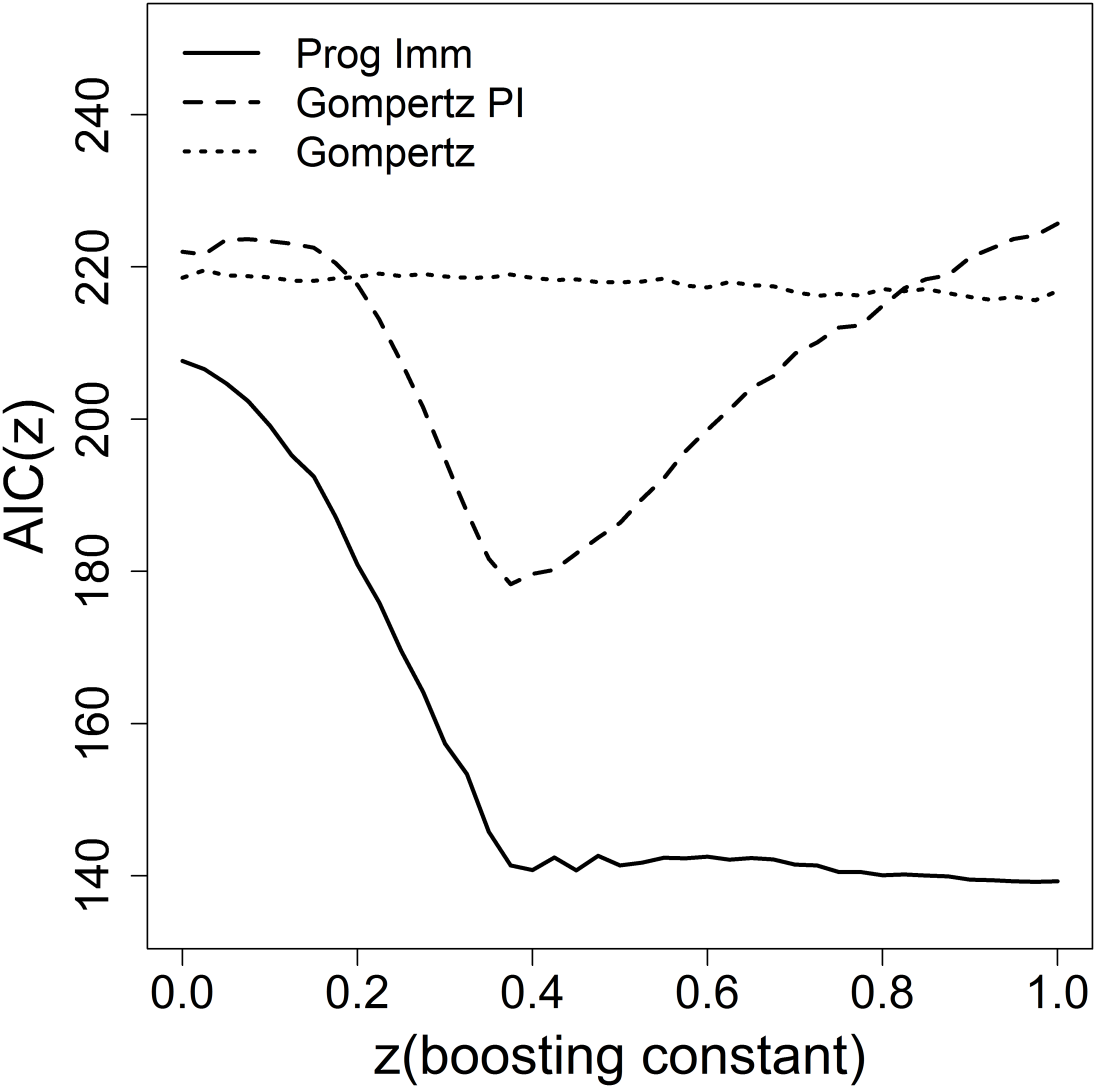
The natural history of VZV in Norway. Pattern of the AIC for some alternative models for the reactivation risk, for different levels of the boosting constant z. The underlying transmission model (Table 2) is based on the synthetic matrix, hypothesis Q_3_, 16.

Results on the imperfect boosting case are summarized in Fig 4, which reports the pattern of the AIC as a function of z for three different models, namely the progressive immunity model, the Gompertz model, and the model combining the senescence and the progressive immunity effects (termed “Gompertz-PI” in Table 1). Fig 4 highlights the following facts. First, the progressive immunity model out-performs other models for all strictly positive values of z, and not only in the perfect boosting scenario. Second, the AIC of the progressive immunity model is essentially flat i.e., the model selection procedure is unable to effectively discriminate between the corresponding models for values of z between 40% and 100%, but it dramatically degrades for z below 40%. Taking the progressive immunity model as a reliable explanation of HZ data, the latter result indicates that a substantial degree of boosting exists, but also that a large uncertainty still remains about its magnitude, which is difficult to pinpoint just from HZ incidence data [10]. Third, the Gompertz model—which fits data poorly compared to the progressive immunity model—shows a flat pattern of the AIC. This is not surprising given the evidence of exponential increase in the HZ data beyond age 40 (when the senescence effect suddenly activates), which makes an exponential risk beyond age 40 effective in explaining the increasing portion of HZ data regardless of whether individuals are boosted fast (high z values) or not (low z values). Last, the model including the senescence and progressive immunity factors exhibits a sharp minimum for z close 0.4 i.e., close to the lower bound of the window of z values selected by the progressive immunity model.

Further results, including bootstrap inference about reactivation parameters are reported in the ESM.

Focusing on the subset of progressive immunity models providing a high quality fit to HZ data (i.e. the models with z in excess of 40%), the boosting constant z and the progressive immunity factor g resulted to be highly correlated (Bravais-Pearson correlation > 99%, see the ESM), namely the higher the boosting intensity the lower is the protective effect of the progressive immunity mechanism which is needed to reproduce the given HZ incidence curve. The effectiveness of boosting, as summarized by the effective reproduction numbers of boosting throughout the different boosting episodes, is shown in Table 5 for the two levels (z = 0.4, z = 1.0) bounding the region of values of the boosting constant which are consistent with HZ data. For z = 1, exogenous boosting is a fast process, where a typical varicella infective individual, besides causing a new varicella infection case (as stated by R_E_), is also able to generate a substantial number of boosts (about 5 overall), while for z = 0.4 little less than two boosting events are still generated.

**Table 5:** Reproduction numbers of boosting.

Boosting episode→	1	2	3	4	5	6	7+	total
z = 0.4	0.79	0.59	0.30	0.12	0.04	0.01	0.00	1.84
z = 1.0	0.94	0.99	0.92	0.74	0.51	0.31	0.30	4.72

The natural history of VZV in Norway. Effective reproduction numbers of boosting (at endemic equilibrium) by boosting episode (columns) for the best fitting progressive immunity models resulting for two different levels (z = 0.4, z = 1) of the boosting constant z (rows). The underlying transmission model is based on by the synthetic matrix, hypothesis Q_3_, 16.

## Discussion

The introduction of varicella vaccination is a debatable subject in European public health. This is due to the fear of the increase of natural HZ following mass immunization that is predicted by most VZV mathematical models based on Hope-Simpson’s EBH [14 –21]. Unfortunately, empirical evidence on this phenomenon in currently vaccinating countries is still controversial [38–41]. Moreover, this phenomenon is difficult to assess due to the presence of confounding effects, such as the increase in HZ incidence observed in various settings prior to the introduction of varicella vaccination (see e.g., [42]), for which the first explanations are fairly recent [43]. Therefore, it is critical, before decisions about immunisation against varicella and HZ are taken, to carefully characterise the local VZV epidemiology, and the consequent potential impact of different immunisation programmes.

In this study we have used mathematical models for VZV transmission and reactivation in combination with available age-structured data on contact patterns, varicella sero-prevalence and herpes zoster incidence, to reconstruct the natural history of VZV in Norway. Results about varicella transmission indicate a somewhat large variation in R_0_ (between 3.7 and 5.0) as a consequence of the broad range of hypotheses made on contact and transmission patterns. This is mirrored into a corresponding variation of the critical coverage (between 73% and 80.0%). As for varicella force of infection the resulting differences are more neatly ascribable to the structural differences in the contact matrices considered. On the one hand, synthetic matrices yield a marked peak during the pre-school period (ages 3–5), with a rapid decline thereafter and a relapse during the childbearing period. On the other hand, Polymod-type [26] matrices yield a much lower peak but the FOI persists at a high level during the overall pre-school and primary school period (age 3–12 years); moreover the peak during childbearing is higher than implied by synthetic matrices and more well-defined.

As regards VZV reactivation, the progressive immunity mechanism [32] explains Norway age-specific HZ incidence data far better than concurring models. As for exogenous boosting, our findings based on a grid analysis of the role of the boosting constant clarify that the joint estimation of boosting and of the reactivation rate under the progressive immunity hypothesis is made complicate due to over-parametrization occurring at high levels of boosting, which makes any level of the boosting constant in excess of 40% consistent with HZ data. On the other hand there is a robust finding, namely that levels of boosting below 40% are not borne out by data. This result brings population-level evidence about the magnitude of exogenous boosting, particularly about the conjecture that a certain degree of CMI boosting must exist although not necessarily at the same rate as the force of infection [10], as instead commonly postulated in the modelling literature [14,15,16,20]. The use of the reproduction numbers of boosting proposed here, allows to quantify the importance of boosting, indicating that when the boosting magnitude is supposed to be maximal (i.e., equal the force of varicella infection), the number of boosting events generated by a typical varicella infective subject is high (about five), but it remains non-negligible also when even the boosting constant is set at its minimum level (i.e., z = in the region of 40%) consistent with HZ data.

Compared to other modeling studies of the natural history of VZV, the present results indicate that varicella transmission in Norway is slower than in the Netherlands and Belgium, but faster than in Central and Southern Europe countries, and the UK [2,28]. Compared to Finland, the varicella FOI in Norway seems to be higher during pre-school (age 3–5 years), lower during primary school (6–12 years), and essentially aligned thereafter. The relapse of the FOI during childbearing, which seems to be a robust phenomenon of varicella transmission across Europe [28], is confirmed for Norway in the present study as well as by independent estimates based on mixture modelling of antibodies data [44]. Overall, estimates of varicella FOI for Norway based on Polymod-type matrices are consistent with those found by studies using Polymod matrices for other European countries [28].

As for HZ, the estimates of reactivation parameters by the progressive immunity model are broadly consistent with those found for Finland [32]. Interestingly, Norway seems to resemble with Finland by showing a sharply lower HZ incidence at most ages compared to Italy and the UK (see Fig 1 in the ESM). This might be suggestive of the presence of a higher exogenous boosting intensity as a specific feature of Nordic countries, as also consistent with the above-described finding on varicella transmission.

Our estimates of varicella transmission and reactivation will be taken, jointly with available contact matrices, as inputs of VZV dynamic models to be used to evaluate the impact of different vaccination options, in order to inform the current discussion on the introduction of varicella and HZ immunization in Norway. In relation to this, our results indicate some issues that will need careful consideration during modelling of vaccination strategies. On one hand, there is evidence of a non-negligible proportion of varicella susceptible individuals among adults, who are at risk of serious varicella disease and congenital varicella syndrome in their offspring [45], especially in consideration of the increasing force of infection during childbearing ages. On the other hand, the predicted higher level of the FOI during the childbearing period jointly with the present findings on re-exposure, suggest that childbearing ages might be important for CMI boosting, so that the suppressive effect due to varicella immunisation should be carefully considered.

Concerning the limitations of this work, the present reconstruction of VZV natural history in Norway was based on serological data documenting past experience of varicella infection, and HZ incidence data, using mathematical modelling relying on available contact matrices. As for the latter point, we compared the results provided by synthetic contact matrices, available for Norway after [25], with those based on diary-informed contact matrices [26], by borrowing Polymod type contact matrices from Finland. The resulting differences for the predicted shape of the force of infection across age are indicative of the presence of model-based uncertainty in transmission which is larger than the statistical uncertainty surrounding estimates from a given matrix, and therefore worth considering in the modeling of the impact of vaccination. Similarly, though the progressive immunity model allows an excellent fit of HZ data, our analyses indicate the possibility of substantial uncertainty about the magnitude of exogenous boosting, which also will be worth considering in future dynamic modeling. Moreover, the hypothesis adopted here that the boosting factor is age-independent must be considered as a simplifying departure point, worth to extend in future studies, perhaps under a different framework for reactivation.

Other critical points lie in the parametrization of reactivation. From this viewpoint, since the progressive immunity formulation is simple (i.e., separable) and parsimonious, it represents a natural “null” hypothesis to be grounded against more refined formulations. For example, in the progressive immunity model the relationship between the reactivation risk and the number of boosting events is represented by an exponentially declining function, therefore potentially going to zero for a large number of boosts. This is an approximation, given that this dependency in reality must have an upper bound in VZV-specific immunity. Similarly the growth in the risk after the last boost is hardly age-independent, rather it is possibly affected by the age at which the last boost occurred. These realistic complications might be worth considering in the future, provided issues of over-parametrization can be properly handled.

More important, our formulation relied on the EBH. Though evidence in favour of the EBH from a number of different investigations, i.e. immunological [8], field [7,2], modelling studies based either on population data [6] or experimental viro-immunological data [9], and meta-analyses [10], has gradually accumulated, suggesting that some exogenous boosting must exist [10], it should be acknowledged that the measurement of its magnitude is probably still inaccurate [10].

Moreover, negative or opposite evidence also exists [46,47]. Other studies [9] suggest that a key role might be played by endogenous forms of boosting, which is major knowledge gap about immunity to HZ, and for which only two modelling studies exists so far [9,48]. The relative importance of exogenous vs endogenous boosting might play a critical influence on the estimated impact of varicella vaccination, and undermine the usefulness of models including exogenous boosting only. Therefore, although the present work has added new population-based insight on the magnitude of exogenous boosting, further progress in modelling VZV reactivation and related implications for vaccination strategies would require substantial progress in the immunologically-based measurement of boosting, both exogenous and endogenous, an area which is still rather under-developed.

## Supporting information

S1 File The mathematical model for VZV transmission and reactivation, and further results. This file reports full details about the mathematical model for VZV transmission and reactivation used in the manuscript, and a number of further results.
(PDF)
