# Supplementary material for: The natural history of varicella zoster virus infection in Norway: Further insights on exogenous boosting and progressive immunity to herpes zoster
Source: PLoS One. 2017 May 18;12(5):e0176845. doi: 10.1371/journal.pone.0176845 (PMC5436649; doi:10.1371/journal.pone.0176845)
Supplement: S1 File — This file reports full details about the mathematical model for VZV transmission and reactivation used in the manuscript, and a number of further results. (PDF) [file pone.0176845.s001.pdf]

Electronic supplementary materials to manuscript:

The natural history of varicella zoster virus infections in Norway: further insights on exogenous boosting and progressive immunity to Herpes Zoster.

Luigi Marangi[1], Grazina Mirinaviciute[2], Elmira Flem[2], Gianpaolo Scalia Tomba[3], Giorgio Guzzetta[4], Birgitte Freiesleben de Blasio[5,2], Piero Manfredi[1].*

[1] Department of Economics and Management, University of Pisa, Pisa, Italy;

[2]Norwegian Institute of Public Health, Oslo, Norway;

[3] Mathematics Department, University of Rome Tor Vergata, Rome, Italy;

[4] Kessler Foundation, Trento, Italy;

[5] Oslo Centre for Biostatistics and Epidemiology, Department of Biostatistics, Institute of Basic Medical Sciences, University of Oslo, Oslo, Norway, and Norwegian Institute of Public Health, Oslo, Norway.

The mathematical model for VZV transmission and reactivation: equations and parameters.

The mathematical model considered (Figure 1 of main text) describes the pre-vaccination endemic equilibrium of VZV in a demographically stationary population, i.e. a population with invariant total size and age distribution allowed by a constant inflow B of births per year and by a time invariant life-table. Besides the introduction of the state of maternal antibody protection, the model equations are essentially analogous to those used in Guzzetta et al. (2013), which in turn extended a model for VZV originally proposed by Karhunen and colleagues (Karhunen et al 2010). The corresponding mathematical system is a mixed ordinary and partial differential equations system, with integral boundary conditions. The system equations and the related boundary conditions are reported in Tab. 1 below, where variables a and τ denote chronological age and duration since the last re-exposure (to VZV) event, respectively. In particular the functions M(a), S(a), I(a) represent the absolute (i.e., non-normalised) densities of individuals having chronological age a which – at the endemic state - are protected by maternal antibodies, susceptible to varicella infection, and varicella infective, respectively, while the functions $HZS_i(a,\tau)$ (i=1,2,3,..) represent

the absolute densities of individuals having chronological age *a*, who experienced i re-exposure events of which the latter τ time units before, at age (a-τ).

Equations	*Boundary conditions*
$M'(a) = -(\mu(a) + \omega(a))M(a)$	$M(0) = B - S(0)$
$S'(a) = \omega(a)M(a) - (\mu(a) + \lambda(a))S(a)$	$S(0) = \left(\frac{1}{2}\right)\int_0^{\infty} S(a)m(a)da$
$I'(a) = \lambda(a)S(a) - (\mu(a) + \gamma)I(a)$	$I(0) = 0$
$\left(\frac{\partial}{\partial a} + \frac{\partial}{\partial \tau}\right)HZS_1(a,\tau) = -(\mu(a) + z\lambda(a) + \rho_1(a,\tau))HZS_1(a,\tau)$	$HZS_1(a,0) = \gamma I(a)$
$\left(\frac{\partial}{\partial a} + \frac{\partial}{\partial \tau}\right)HZS_2(a,\tau) = -(\mu(a) + z\lambda(a) + \rho_2(a,\tau))HZS_2(a,\tau)$	$HZS_2(a,0) = z\lambda(a)\int_0^a HZS_1(a,\tau)d\tau$
...	...
$\left(\frac{\partial}{\partial a} + \frac{\partial}{\partial \tau}\right)HZS_i(a,\tau) = -(\mu(a) + z\lambda(a) + \rho_i(a,\tau))HZS_i(a,\tau)$	$HZS_i(a,0) = z\lambda(a)\int_0^a HZS_{i-1}(a,\tau)d\tau$
...	...
$\frac{d}{da}HZI(a) = \sum_i \int_\tau \rho_i(a,\tau)HZS_i(a,\tau)d\tau - (\mu(a) + \gamma_{HZ})HZI(a)$	$HZI(0) = 0$
$\frac{d}{da}HZR(a) = \gamma_{HZ}HZI(a) - \mu(a)HZR(a)$	$HZR(0) = 0$

Tab. 1. (Equilibrium) Equations for the model for VZV transmission and reactivation and corresponding boundary conditions.

As regards demographic parameters, B denotes the inflow of births per unit time, while $\mu(a)$ and m(a) denote age-specific mortality and fertility rates, respectively. As for epidemiological parameters, $\omega(a)$ is the rate of decay of maternal antibody protection, which is assumed to obey a type-1 form i.e., maternal antibody protection has a fixed duration D_M; λ(a) is the force of varicella infection at equilibrium, which has the piece-wise constant form reported in the main text: $\lambda(a) = \sum_{j \in G} q_{ij}\, C_{ij}\, \frac{I_J}{N_J}, \;\; a_{i-1} < a < a_i$; γ is the rate of recovery from varicella infection; z is a constant scaling the force of CMI boosting with respect to the force of infection according to $\lambda_B^{(i)}(a) = z \cdot \lambda(a)$, $\rho_i(a,\tau)$ is the age-specific reactivation rate per susceptibility stage, and γ_{HZ} is the rate of recovery from active zoster disease.

In particular the age-density $HZS_i(a)$ of susceptible individuals who experienced i re-exposure episode irrespective of the time elapsed since last-exposure is given by:

$$HZS_i(a)=\int_0^a HZS_i(a,\tau)d\tau$$

The model parameters are summarised in Tab. 2.

Parameters	***Unit***	**Usage**	***Range or value***	***Source***
$\mu(a)$, , age-specific mortality rate	yr^{-1}	Fixed		Eurostat, Norway life table 2000-2010
m(a), age-specific fertility rate	yr^{-1}	Fixed		Eurostat, Norway fertility data 2000-2010
D_M, duration of maternal antibodies protection	*yr*	Assigned by preliminary fit to serological data	0.5yr	This paper.
$C=[C_{ij}]$=contact matrix, whose elements C_{ij} represent the average number of contacts between individuals aged (i,j) per unit of time	yr^{-1}	Fixed	---	Norway matrix of all contacts (Fumanelli et al 2013); Polymod matrices for Finland (Mossong et al 2008)
$q_{ij}(a_c)$= age-specific varicella transmission coefficient per contact between individuals aged (i,j) and separation age a_c	-	Fitted to Norway serological data (see main text).		This paper.
γ = varicella recovery rate	yr^{-1}	Fixed	$1/\gamma$ = 7 days	Heynman 2004.
$\rho_i(a,\tau,a_0)$ = VZV reactivation rate of development of natural HZ for individuals in age group i	yr^{-1}	Fitted to Norway HZ incidence data under the forms in Tab. 2 of main text.		This paper
a_0 = age at which the exponential increase in the reactivation hazard initiates	*yr*	Assigned by preliminary data analyses (see main text)	a_0 =40 yr.	This paper
γ_{HZ}= zoster recovery rate	yr^{-1}	Fixed	$1/\gamma_{HZ}$= 11 days	Heynman 2004.

Tab. 2. Summary information about model parameters (definitions, units, sources).

Demographics

By integrating with respect to τ and adding the equations in Tab. 1, one gets the equation for the age distribution of the total population n(a):

$$n'(a)=-\mu(a)n(a)$$
$$n(0)=B$$

The previous equation describes a demographically stationary population determined by a constant inflow B of births per unit of time, coupled with a time invariant life table with force of mortality μ(a). An integration yields:

$$n(a) = Be^{-\int_0^a \mu(x)dx} = Bp(a)$$

where p(*a*) denotes the survival (to mortality) function, representing the probability that a newborn individual survives up to (exact) age *a*. The number of yearly births B is computed for simplicity as:

$$B = \left(\frac{1}{2}\right)\int_0^\infty n(a)m(a)da$$

where (1/2) represent the proportions of female individuals at each age, on the assumption that the sex-ratio at birth is 50% and applying a unique life-table to individuals of both sexes. Fertility rates are scaled up to one to ensure population stationarity through the condition:

$$\left(\frac{1}{2}\right)\int_0^\infty p(a)m(a)da = 1$$

Births of susceptible individuals

In particular, yearly births are split into those who are born susceptible to varicella and whose who are born immune (by acquired maternal antibodies) as B=M(0)+S(0), where:

$$S(0) = \left(\frac{1}{2}\right)\int_0^\infty S(a)m(a)da$$

Age-specific incidence of herpes zoster

The absolute HZ incidence among individuals aged *a*, which we denote by *HZC(a),* is obtained by taking the absolute incidence among i-type HZS susceptible individuals, given by $HZC_i(a,\tau) = \rho_i(a,\tau)HZS_i(a,\tau)$, and then summing over number of re-exposures and integrating over time elapsed since the last exposure:

$$HZC(a) = \sum_{i=1}^{\infty}\int_0^a \rho_i(a,\tau)HZS_i(a,\tau)d\tau$$

References about the VZV model

Karhunen M, Leino T, Salo H, Davidkin I, Kilpi T, Auranen K. Modelling the impact of varicella vaccination on varicella and zoster. Epidemiol Infect. 2010;138:469–81. doi:10.1017/S0950268809990768.
Guzzetta G, Poletti P, Del Fava E, Ajelli M, Scalia Tomba GP, Merler S, et al.Hope-Simpson's progressive immunity hypothesis as a possible explanation for herpes zoster incidence data. Am. J. Epidemiol 2013; 177: 1134–42. (doi:10.1093/aje/kws370).

Further details and references about estimation of transmission parameters

As detailed in the main text, thanks to the assumption that active zoster cases do not contribute to the force of infection of varicella it is possible to estimate the transmission part of the model by an MSIR model. For each transmission model considered in the main text, the estimation of transmission parameters was carried out by the standard approach based on (i) formal resolution of the equations for epidemiological variables at endemic equilibrium over 1-year age groups, based on the assumption that the endemic FOI is piece-wise constant over the different age groups, (ii) maximisation of the Bernoulli log-likelihood of serological data with respect to the transmission parameters conditional on the assumed age-grouping and under the restraint that the ensuing FOI in turn fulfils its equilibrium equation. This approach is nowadays standard and readers can refer to the many publications available, for example Farrington et al 2001, Wallinga et al 2006, Goeyvaerts et al 2010, Iozzi et al 2010, etc.
In particular the MSIR models considered in the paper were fitted by including all ages in the serological likelihood.

References on FOI estimation.

Farrington, C. P., Kanaan, M. N., & Gay, N. J. (2001). Estimation of the basic reproduction number for infectious diseases from age-stratified serological survey data. Journal of the Royal Statistical Society: Series C (Applied Statistics), 50(3), 251-292.
Wallinga J, Teunis P, Kretschmar M. Using social contact data to estimate age-specific transmission parameters for infectious respiratory spread agents. Am J Epidemiol. 2006;164(10):936–944.
Goeyvarts N, Hens N, Ogunjimi B, Aerts M, Shkedy Z, et al. (2010) Estimating infectious disease parameters from data on social contacts and serological status. Appl Statist 59(2): 255–277.
Iozzi, F., Trusiano, F., Chinazzi, M., Billari, F. C., Zagheni, E., Merler, S. et al. (2010). Little Italy: an agent-based approach to the estimation of contact patterns-fitting predicted matrices to serological data. PLoS Comput Biol, 6(12), e1001021.
Melegaro A, Jit M, Gay N, Zagheni E, Edmunds WJ (2011) What types of contacts are important for the spread of infections? Using contact survey data to explore European mixing patterns. Epidemics 3(3–4): 143–151.
Santermans E, Goeyvaerts N, Melegaro A, Edmunds WJ, Faes C, Aerts M, Beutels P, Hens N, The social contact hypothesis under the assumption of endemic equilibrium: Elucidating the transmission potential of VZV in Europe, Epidemics 11 (2015) 14–23.

Age-patterns in HZ incidence in European countries.

A characteristic of herpes zoster already noted in the cited study by Nardone et al (2007) is the wide variation in age –specific incidence across European countries. The graph below shows the

point remarked in the Discussion i.e., that Norway seems to cluster with Finland in showing a sharply lower HZ incidence by age compared to Italy and the UK (see also Poletti et al 2013, Guzzetta et al 2013).

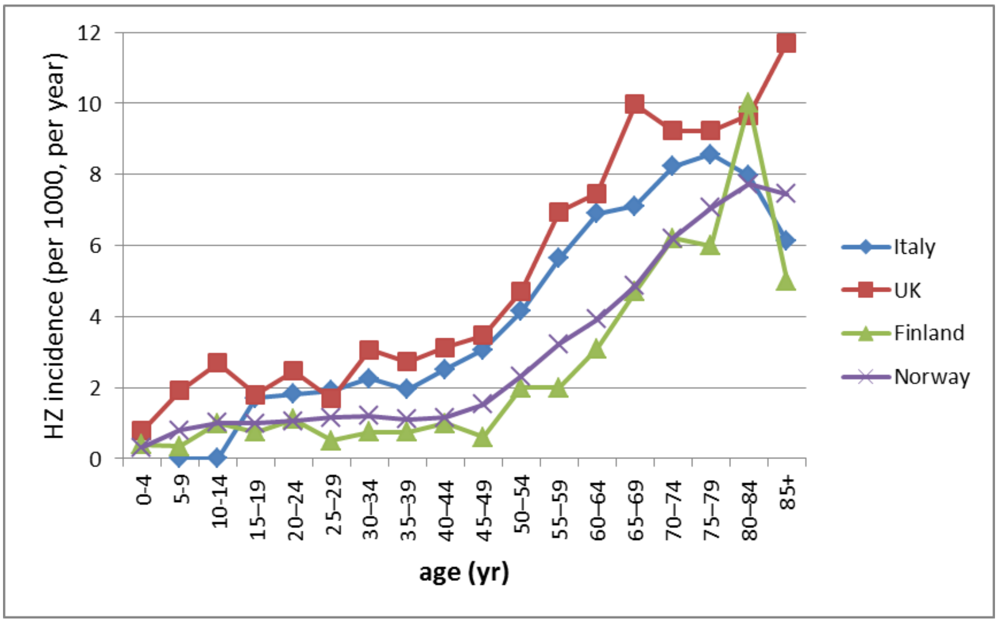

Fig. 1. Age-specific HZ incidence profiles in 4 European countries.

References on HZ incidence in European countries

Nardone A, de Ory F, Carton M, Cohen DI, Van Damme P, Davidkin I, et al. The comparative sero-epidemiology of varicella zoster virus in 11 countries in the European region. Vaccine 2007;25:7866–72. doi:10.1016/j.vaccine.2007.07.036.

Poletti P, Melegaro A, Ajelli M, Del Fava E, Guzzetta G, Faustini L, et al. Perspectives on the Impact of Varicella Immunization on Herpes Zoster. A Model-Based Evaluation from Three European Countries. PLoS ONE 2013;8:e60732. doi:10.1371/journal.
pone.0060732.s001.

Guzzetta G, Poletti P, Del Fava E, Ajelli M, Scalia Tomba GP, Merler S, et al.Hope-Simpson's progressive immunity hypothesis as a possible explanation for herpes zoster incidence data. Am. J. Epidemiol 2013; 177: 1134–42. (doi:10.1093/aje/kws370).

Selection of the chronological age threshold a_0.

As stated in the Methods section of the main text, we also attempted to better identify the age a_0 above which the age component of the reactivation rate starts increasing rapidly. In relation to this we have run some preliminary experiments by fitting to HZ incidence data the reactivation models reported in the main text (Table 1) over a grid of values of a_0 ranging between 30 and 60 years of age, with a step equal to one year. This analysis has showed that for both the progressive immunity, the baseline and the Gompertzian model there is a rather narrow window of values surrounding the value a_0 = 40 yrs which is compliant with HZ data. This is illustrated in Fig. 2 below which reports, for each value of a_0 in the grid, the corresponding values of the AIC of the best progressive immunity model based on the synthetic matrix, hypothesis Q3, 16 (see main text, Table 2 and Table 4). Following these findings, in the subsequent analyses reported on the Results section of the main text we retained for simplicity a_0 = 40 yr.

Though one might expect that senescence-related effects should arise later - for example in Karhunen et al (2010) the value a_0 =60 yr was also considered – it should be recalled that the Gompertzian force, representing ageing, becomes the key determinant of human mortality already above age 30.

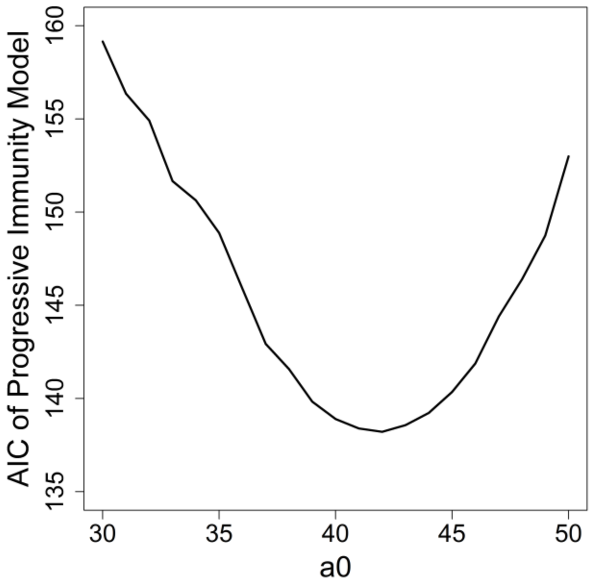

Fig. 2. The natural history of VZV in Norway. Trend in the AIC of the best progressive immunity model (under the case of perfect boosting, z=1) for different levels of the threshold age a_0. The underlying transmission model is based on the synthetic matrix, hypothesis Q_3, 16.

References

Karhunen M, Leino T, Salo H, Davidkin I, Kilpi T, Auranen K. Modelling the impact of varicella vaccination on varicella and zoster. Epidemiol Infect. 2010;138:469–81. doi:10.1017/S0950268809990768.

Fitting HZ incidence by excluding the very old

The progressive immunity model is the only one, among the various models for the reactivation rate considered in the manuscript (see Table 1 of the main text), that allows to capture well the drop in HZ incidence among very old individuals. Nonetheless, the progressive immunity model continues to perform better than alternative models even disregarding the decline in incidence among very old individuals (Guzzetta et al 2013), as also noted in the main text. To check this for the Norwegian case, we re-fitted the various models considered to HZ incidence data truncated at age 84. The ensuing results are reported below in Tab. 3 and Fig. 3 for the hypothesis of perfect boosting, under the transmission model based on the synthetic matrix, hypothesis Q_3, 16, showing that the progressive immunity model still performs largely better than alternative models in terms of model selection measures, with differences in AIC still largely in excess of 6. Analogous results hold for other hypotheses on transmission as well as for different values of the boosting constant allowing a high quality fit to HZ incidence data. Obviously, the extent of the differences in AIC between the progressive immunity and the concurrent models are much smaller than in Table 4 of the main text, due to the removal of the right portion of the HZ incidence curve, where the progressive immunity model gains in terms of fit.

	ρ_0	ϑa	ϑ_τ	g	AIC
Progressive Immunity	0.00154	0.05302	0.03846	0.64098	120.9
Baseline	0.00093	0.03923	0.02185	1.00000	136.1
Gompertz	0.00125	0.05305	0.00000	1.00000	132.9

Tab. 3. The natural history of VZV in Norway. Fit to HZ incidence data performed including age 0-84 only: parameter estimates and model selection measures for a number of models for VZV reactivation.The fit is carried out under the hypothesis of perfect boosting (z=1). The underlying transmission model is based on the synthetic matrix, hypothesis Q_3, 16.

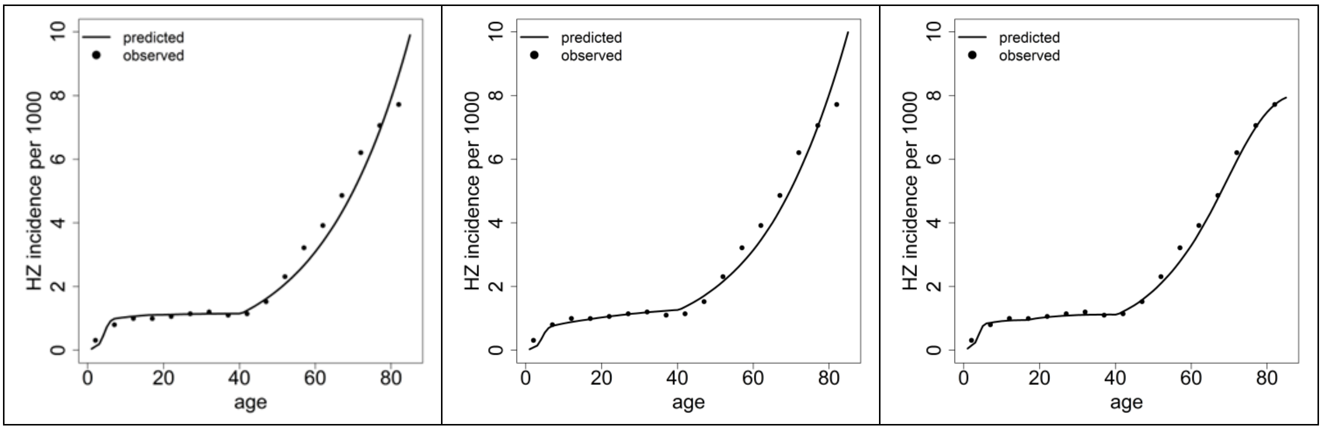

Fig. 3. The natural history of VZV in Norway. Fit to age-specific HZ incidence on ages 0-84(per 1000 population, per year) by some of the alternative models for VZV reactivation considered in the main text. The fit was carried out under the hypothesis of perfect boosting (z=1). Left: the best Gompertzian model; centre: the best baseline model; right: the best progressive immunity model. The underlying transmission model is based on the synthetic matrix, hypothesis Q_3, 16.

Fitting HZ incidence over 1-year age groups

The results on estimation of the VZV reactivation rate reported in the main text were based on age-specific HZ incidence data grouped by five years age groups, mainly for sake of comparison with previous work (Guzzetta et al 2013). Given that HZ data for Norway were available on a 1-year age scale, we also carried out the fit for this case, observing negligible differences in results, with the progressive immunity model still outperforming other models for reactivation. Just for completeness we report here the graphic comparison between the observed HZ incidence and the HZ incidence (per 1000) predicted by the best-fitting progressive immunity model based on the synthetic matrix, hypothesis Q3, 16.

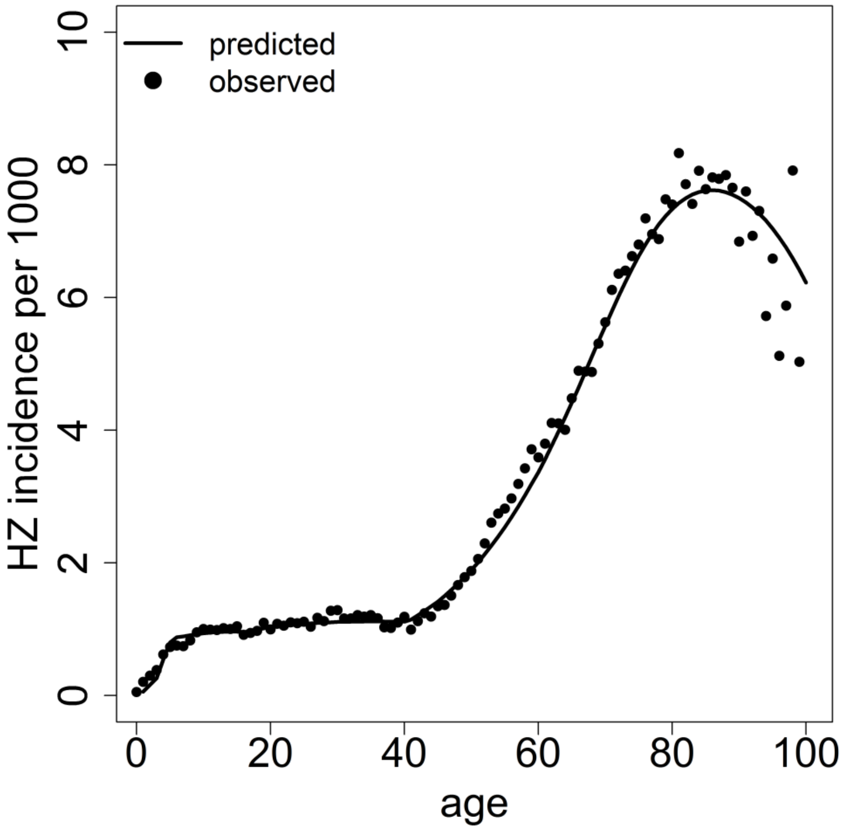

Fig. 4. The natural history of VZV in Norway and the perfect boosting hypothesis (z=1). Fit of the progressive immunity model to age-specific HZ incidence data by 1-year age group (per 1000 population, per year). The underlying transmission model is based on the synthetic matrix, hypothesis Q_3, 16.

More about exogenous boosting and progressive immunity

The graph in Figure 3 of the main text suggests the issues of overfitting and identifiability which would arise if the boosting constant z and the progressive immunity factor *g* are jointly estimated: our grid analysis of the boosting constant z indicates that for values of z in excess of 40% the progressive immunity model always provides an accurate fit to HZ incidence data but the various models considered are indistinguishable according to standard measures of model selection as the AIC. This result in particular means that the values of z and of the resulting estimates of the progressive immunity factor g are highly (positively) correlated (Fig. 5), with a Pearson correlation coefficient around r=0.99). The vslight departures from the trend seem to essentially be the consequence of the fact that for each level of the boosting constant z we fully refitted the model (that is the entire 4-dim parametric vector of the progressive immunity schedule is refitted) yielding slight oscillations in the estimates, given the tolerance level of the estimation problem). Fig. *6* offers a graphic comparison of the fit between the full boosting case (z=1, right panel) and the value z=0.4 (left panel), which is close to the lower bound of the set of values of the boosting constant supplying high quality fits to HZ incidence data.

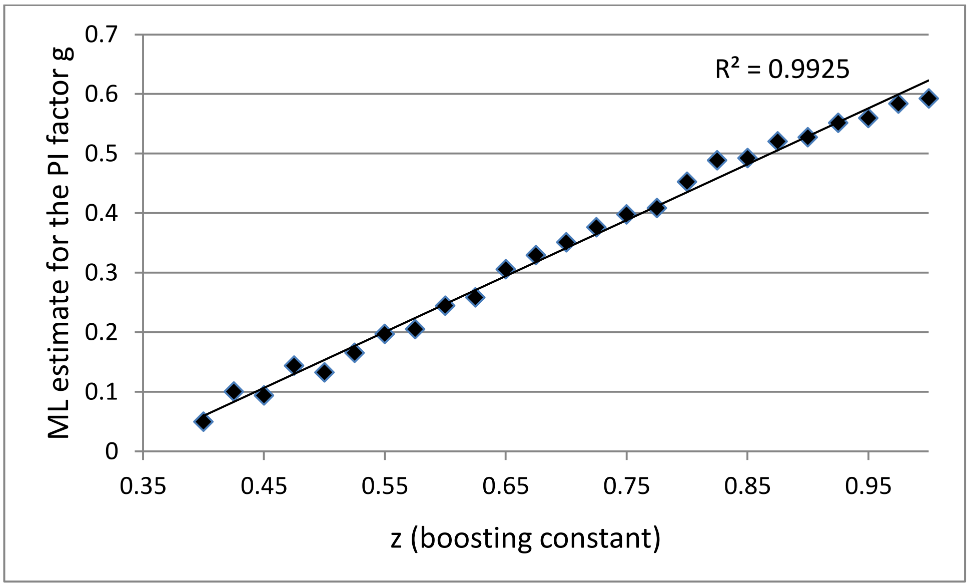

Fig. 5. The perfect boosting hypothesis (z=1). Scatterplot of the ML estimates of parameter g tuning the intensity of the progressive immunity mechanism drawn vs the corresponding levels of the boosting constant z, for levels of z allowing a high quality fit to HZ data. The underlying transmission model is based on the synthetic matrix, hypothesis Q_3, 16.

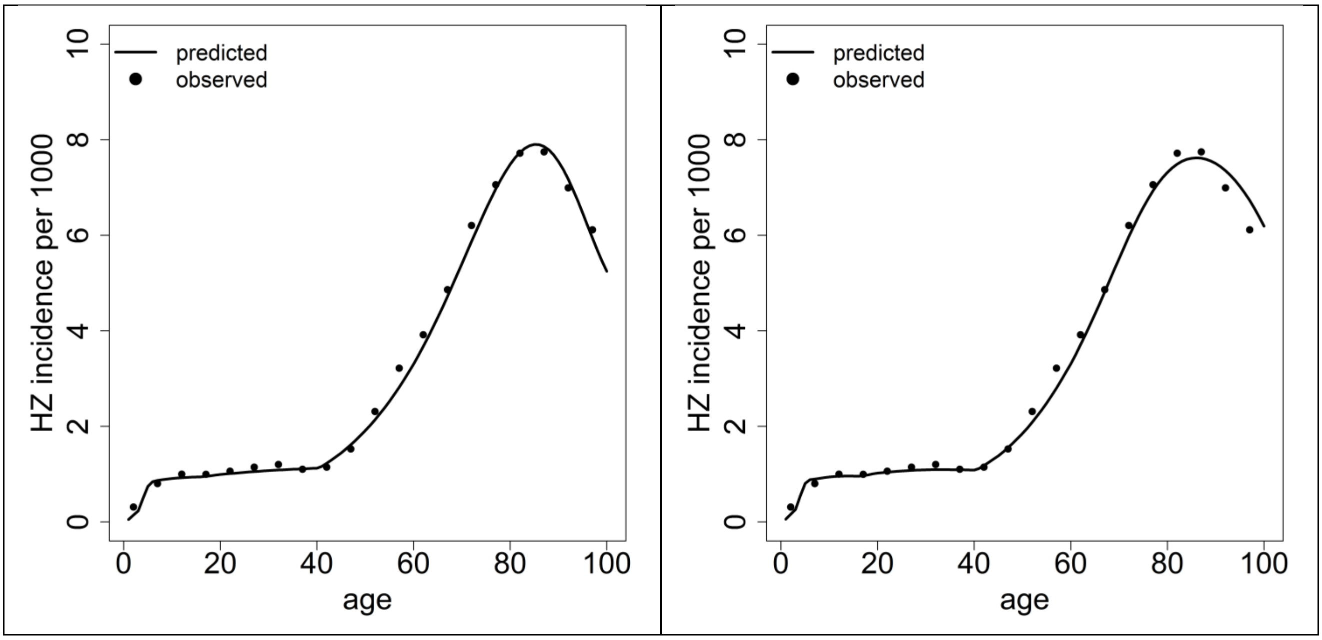

Fig. 6. The natural history of VZV in Norway. Graphic comparison of the fit to HZ incidence data by the progressive immunity model for z=0.4 (left) and for z=1.0 (right). The underlying transmission model is based on the synthetic matrix, hypothesis Q_3, 16.

Bootstrap inference about the reactivation model.

Consistently with the two-stage estimation approach presented in the Methods, bootstrap inference about the parameters tuning the VZV reactivation rate was carried out as follows: (i) the uncertainty directly related with reactivation parameters given the force infection (stage 2), was assessed by parametric bootstrap by resampling for each age class considered from the Poisson distribution with expectation given by the corresponding absolute incidence from the best fitted reactivation model; (ii) the uncertainty related to the estimation of the FOI in the first stage (main text, Table 2) was incorporated by taking the family of forces of infection associated with the

corresponding 95% bootstrap percentile confidence interval of transmission parameters, and refitting, for each of the FOIs considered the model for reactivation. In Tab. 4 below we report the 95% bootstrap percentile confidence interval for the reactivation parameters of the progressive immunity model based on the transmission model Q3, 16 (see main text), under the hypothesis of perfect boosting (z=1) (note that the ML estimates of reactivation parameters are those reported in Table 4 of the main text). The resulting confidence bands are reported in Fig. *7*.

**Parameters→**	ρ_0	ϑ_a	ϑ_τ	g
ML estimates and related 95% CI	*0.00166* *(0.00132, 0.00200)*	*0.05733* *(0.04378, 0.07088)*	*0.03723* *(0.02541, 0.04905)*	*0.59245* (0.45574, 0.72915)

Tab. 4. The natural history of VZV in Norway. ML estimates of the parameters of the progressive immunity model for the perfect boosting case (z=1) with corresponding 95% bootstrap percentile confidence bands (below, in parentheses). The underlying transmission model is based on the synthetic matrix, hypothesis Q_3, 16.

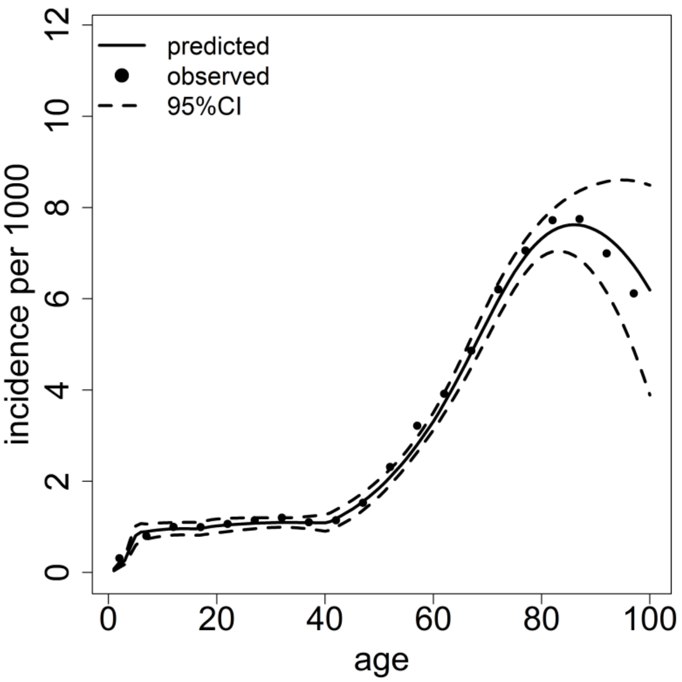

Fig. 7. The natural history of VZV in Norway. Fit of age-specific HZ incidence by the best progressive immunity model with corresponding 95% bootstrap percentile confidence bands under the perfect boosting hypothesis (z=1.0). The underlying transmission model is based on the synthetic matrix, hypothesis Q_3, 16.
